# Phenotypic variation of floral organs in flowering crabapples and its taxonomic significance

**DOI:** 10.1186/s12870-021-03227-8

**Published:** 2021-10-30

**Authors:** Ting Zhou, Kun Ning, Wangxiang Zhang, Hong Chen, Xiaoqing Lu, Donglin Zhang, Yousry A. El-Kassaby, Jian Bian

**Affiliations:** 1 Jiangsu Key Laboratory for the Research and Utilization of Plant Resources, Institute of Botany, Jiangsu Province and Chinese Academy of Sciences (Nanjing Botanical Garden Mem. Sun Yat-Sen), Nanjing, 210014 China; 2 College of Horticulture, Jinling Institute of Technology, Nanjing City, Jiangsu Province 210038 P.R. China; 3 College of Forestry, Nanjing Forestry University, Nanjing, 210037 China; 4 Department of Horticulture, University of Georgia, Athens, GA 30602 USA; 5 Department of Forest and Conservation Sciences, Faculty of Forestry, University of British Columbia, Vancouver, BC Canada; 6 Jiangsu Yufeng Tourism Development Co. Ltd., Yancheng, 224000 China

**Keywords:** Flowering crabapple, Floral organ phenotype, Numerical taxonomy, Ancestor-inclined distribution, Phenotypic variation, Taxonomic significance

## Abstract

**Background:**

In angiosperms, phenotypic variation of floral organs is often considered as the traditional basis for the evolutionary relationship of different taxonomic groups above the species level. However, little is known about that at or below the species level. Here, we experimentally tested the phenotypic variation of *Malus* floral organs using combined methods of intraspecific uniformity test, interspecific distinctness analysis, principal component analysis, Pearson correlation analysis, and Q-type cluster analysis. The ancestor-inclined distribution characteristic analysis of *Malus* species and cultivars floral attributes was also carried out, so as to explore its taxonomic significance.

**Results:**

15/44 phenotypic traits (e.g., flower shape, flower type, flower diameter, ...) were highly consistent, distinguishable, and independent and could be used as the basis for *Malus* germplasm taxonomy. The studied 142 taxa were divided into two groups (A, B) and five sub-groups (A_1_, A_2_, B_1_, B_2_, B_3_), with significantly variable floral phenotypic attributes between groups and within sub-groups. *Malus* natural species were relatively clustered in the same section (series) while homologous cultivars showed evidence of ancestor-inclined distribution characteristics. However, no significant correlation between the evolutionary order of sections (Sect. *Docyniopsis* → Sect. *Chloromeles* → Sect. *Sorbomalus* → Sect. *Eumalus*) and group/sub-groups (B_3_ → B_2_ → B_1_ → A).

**Conclusions:**

Phenotypic variation of floral organs could better explore the genetic relationship between *Malus* taxa. The findings improved our cognition of floral phenotypic variation taxonomic significance under the species level.

## Background

Flowering crabapples (*Malus* spp.) are small trees or shrubs in the rose family, characterized by enchanting flowers, colorful small fruits (≤5 cm), and various growth habits. They are also valued for their wide environmental adaptability, facilitating world-wide prominence as gardens and landscape focal points [1–3]. *Malus* germplasm harbors high level of diversity due to a long period of crossbreeding and natural selection, with steadily growing number of varieties and cultivars compared to their wild ancestors [4–7]. While nearly 1200 *Malus* taxa were documented in Fiala’s “*Flowering Crabapple*” book, approximately 60 are with known pedigree [8]. It is interesting to note that the majority of the recorded cultivars arose from chance seedlings or selective breeding, thus some of their relationships and genealogy remain unclear [8–12].

Flowers, as a unique and highly conserved morphological feature of angiosperms and are often considered as the traditional traits for complex phenotypic identification of different taxonomic groups, as well as evaluating the interplay between evolution and developmental bias [13–15]. Since the information obtained from floral morphological characterizations is consisted of large qualitative and quantitative traits datasets, multivariate analyses are considered to be the most suitable analytical tools for their evaluation [16, 17]. Numerical taxonomy, as one of the multivariate analyses, accelerates the application of systematic taxonomy in plant evolution by quantitatively evaluating the morphological similarity between taxonomic groups [18]. However, the objectivity of the taxonomic results is greatly affected by the selected morphological traits. Recently, in ornamental plant germplasm numerical taxonomy studies, principal component analysis (PCA) is often used to reduce data dimensionality and can be supplemented with one-way analysis of variance (ANOVA) and correlation analysis (R-type cluster analysis: the classification of data objects into similarity groups) [19, 20]. No scientific system has been formed for trait selection. Moreover, taxonomic units of some studies were solely established above the species level, and the conducted analyses were simply limited to the germplasm identification or clustering group division. This resulted in a failure to correctly locate the role of species, as to conduct in-depth discussion of genetic/evolutionary relationship analysis at or below the species level [21–27].

Based on floral organ phenotypes, we performed numerical taxonomy of 142 *Malus* taxa to address the following objectives: 1) establishing a scientific system for *Malus* taxonomic traits selection; 2) revealing the extent of *Malus* floral organs phenotypic diversity; and 3) clarifying the taxonomic significance (genetic or evolutionary relationships) of *Malus* floral variation.

## Results

### Intraspecific uniformity test and interspecific distinctness analysis of floral phenotypic traits

**Fig. 1:**
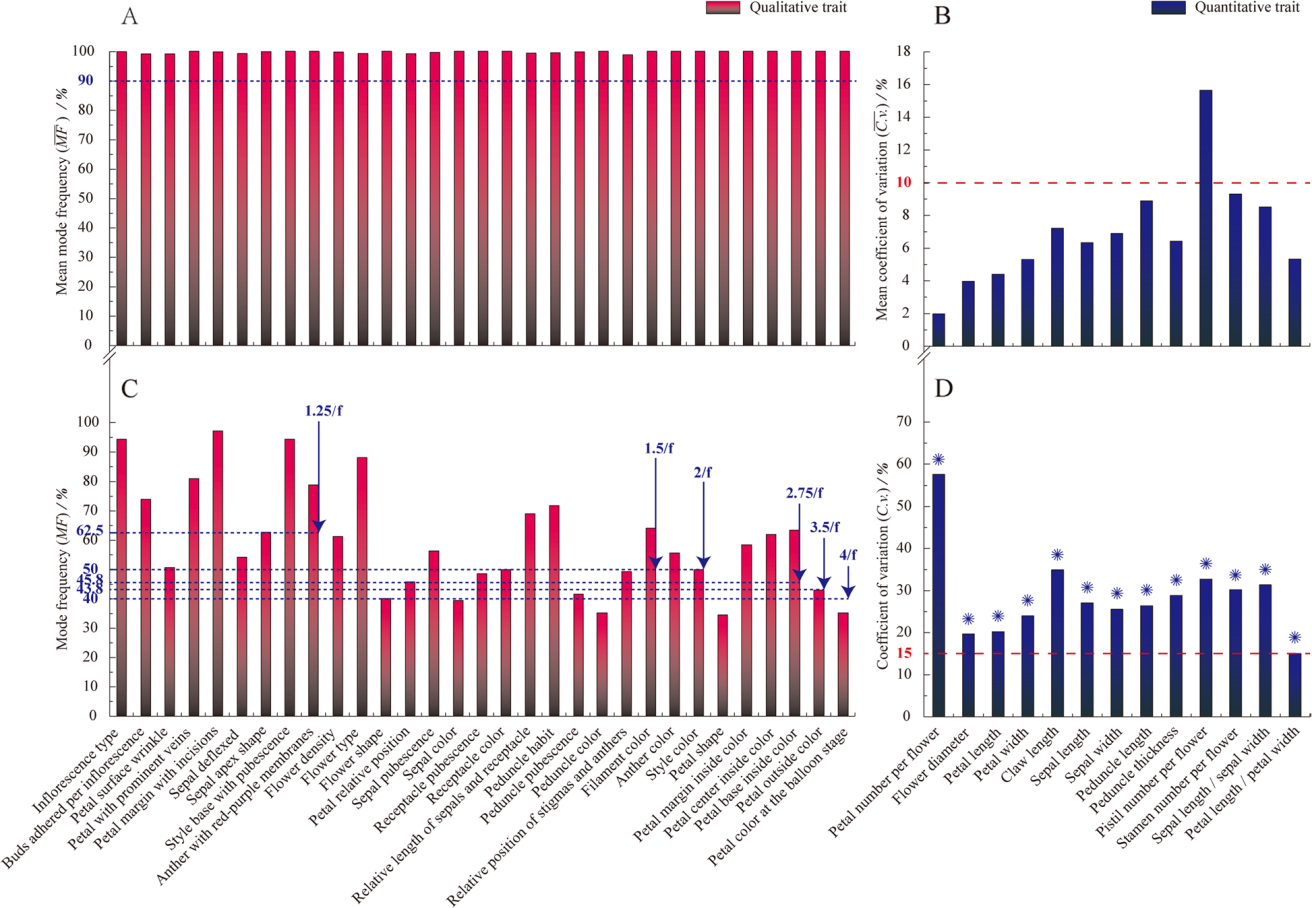
Intraspecific uniformity test and interspecific distinctness analysis of *Malus* floral phenotypic traits. **A** The intraspecific uniformity of floral organs qualitative traits using 90% as the criteria. If $\overline{MF}$≥90%, then the qualitative trait has met the uniformity requirements. **B** The intraspecific uniformity of floral organs quantitative traits using 10% as the criteria. If $\overline{C.v.}$≤10%, then the quantitative trait has met the uniformity requirements. **C** The interspecific distinctness of floral organs qualitative traits using $k\,{1}\left/{f}\right.$ as the reference. *k* is a coefficient that depends on the number of ranks (*f*) of each trait appeared in all taxa. The specific assignment is indicated in the **C**. If $MF\le k\,{1}\left/{f}\right.$, the qualitative trait is more differentiated among all taxa. **D** The interspecific distinctness of floral organs quantitative traits using 15% as the criteria. If *C*. *v*.≥15%, it was considered that the differentiation degree of this trait is high among all the taxa. For more accurate expression, the one-way ANOVA (Tukey’s method) was performed. * represents that the difference of quantitative traits reached a significant level between different *Malus* taxa (*P* < 0.05)

Except for the pistil number per flower, the remaining 43 qualitative and quantitative traits had significant intraspecific uniformity ($\overline{MF}$≥90%, $\overline{C.v.}$≤10%), meeting the requirement for taxa classification (Fig. 1A, B). As for the interspecific distinctness of qualitative traits, it was found that only 15 floral traits (petal surface wrinkle, sepal deflexed, sepal apex shape, flower shape, petal relative position, sepal color, receptacle pubescence, receptacle color, peduncle pubescence, peduncle color, relative position of stigmas and anthers, style color, petal shape, petal outside color, and petal color at the balloon stage) showed a high degree of distinctness among taxa ($MF\le k\,{1}\left/{f}\right.$). The remaining 16 qualitative traits, all had no significant interspecific distinctness, and thus should not be further considered in the analyses. It is worth mentioning that although flower type (variable that reflects the number of petal whorls or petals) was less differentiated among the taxa, still it was retained in this analysis for its high recognition value (Fig. 1C). All 13 quantitative traits had a high degree of distinctness (*C*. *v*.≥15%), and could be used as taxonomic trait candidates (Fig. 1D).

### Principal component analysis of floral phenotypic traits

Based on the 28 floral organs phenotypic traits (16 qualitative and 12 quantitative) selected by the above-mentioned intraspecific uniformity test and interspecific distinctness analysis, principal component analysis was then performed. Taking eigenvalue *λ*> 0.85 as the extraction threshold, a total of 10 principal components mainly consisted of 22 related floral traits were extracted with a cumulative variation contribution of 83.71%, reflecting most of the information of the original floral dataset (Table 1).

**Table 1:** Eigenvalue, contribution rate and cumulative contribution rate of each principal component

Principal Component	PC 1	PC 2	PC 3	PC 4	PC 5	PC 6	PC 7	PC 8	PC 9	PC 10
Load coefficient										
Floral phenotypic traits										
Petal color at the balloon stage	**0.93**	0.11	0.11	−0.05	− 0.08	− 0.01	0.03	− 0.09	− 0.01	0.02
Peduncle color	**0.90**	−0.01	− 0.05	0.08	0.04	0.12	−0.03	−0.04	0.05	0.09
Sepal color	**0.89**	−0.08	0.12	0.11	0.09	0.19	0.08	0.03	0.13	0.04
Petal outside color	**0.89**	0.14	0.10	0.00	−0.11	−0.05	0.09	−0.13	0.01	0.09
Receptacle color	**0.83**	0.22	0.02	−0.02	0.03	0.08	−0.14	0.06	0.16	0.06
Style color	**0.75**	−0.13	− 0.09	0.22	0.07	−0.14	0.05	0.01	−0.23	0.08
Flower diameter	0.11	**0.95**	0.02	0.12	−0.11	−0.06	0.04	0.02	−0.08	0.10
Petal length	0.11	**0.94**	0.02	0.07	−0.07	−0.08	0.07	0.05	0.02	0.11
Petal width	0.04	**0.81**	−0.48	0.06	−0.11	0.05	0.11	0.06	−0.01	0.08
Sepal length	−0.11	0.68	−0.20	−0.33	− 0.09	−0.43	− 0.06	0.05	− 0.04	−0.20
Peduncle thickness	−0.06	0.59	−0.17	0.06	−0.50	0.18	−0.10	0.26	0.02	−0.21
Claw length	0.11	0.58	0.26	0.18	−0.26	− 0.05	−0.11	− 0.34	−0.31	0.21
Sepal width	−0.02	0.56	−0.38	−0.08	− 0.26	0.47	0.03	0.23	0.05	−0.25
Petal length / Petal width	0.09	−0.08	**0.86**	−0.02	0.10	−0.22	−0.11	0.01	0.08	−0.01
Petal shape	0.12	−0.06	**0.79**	0.15	0.04	−0.03	0.13	0.18	−0.09	0.07
Petal relative position	0.05	0.12	**−0.74**	0.30	−0.04	0.10	0.02	0.24	0.13	0.06
Petal number per flower	0.11	0.08	−0.05	**0.94**	0.00	−0.03	0.15	0.06	−0.07	−0.06
Flower type	0.10	0.07	−0.04	**0.93**	0.04	0.15	0.04	0.08	0.01	0.09
Receptacle pubescence	−0.03	−0.20	0.06	0.05	**0.92**	0.02	0.08	0.07	0.03	−0.04
Peduncle pubescence	0.03	−0.14	0.08	−0.01	**0.91**	0.04	0.04	−0.02	0.07	0.12
Sepal length / Sepal width	−0.04	0.11	0.16	−0.07	0.13	**−0.86**	−0.02	− 0.16	−0.17	− 0.06
Sepal apex shape	0.15	−0.04	−0.20	0.08	0.23	**0.74**	0.00	−0.16	−0.11	− 0.05
Peduncle length	0.04	0.08	−0.05	0.12	0.15	−0.04	**0.90**	0.03	0.04	0.16
Pistil number per flower	−0.02	0.10	0.25	0.44	−0.13	0.35	0.53	0.11	0.05	−0.33
Petal surface wrinkle	−0.10	0.12	0.03	0.14	0.01	0.02	0.06	**0.85**	−0.10	0.07
Relative position of stigmas and anthers	0.16	−0.14	−0.11	0.01	0.11	0.09	0.07	−0.08	**0.83**	−0.06
Sepal deflexed	−0.44	0.17	0.10	−0.28	−0.13	− 0.14	−0.36	− 0.11	0.45	0.10
Flower shape	0.26	0.12	0.01	0.02	0.10	0.01	0.11	0.08	−0.04	**0.85**
**Eigenvalue**	5.50	5.11	3.25	2.50	1.86	1.35	1.04	0.99	0.97	0.87
**Contribution rate / %**	19.66	18.26	11.60	8.93	6.64	4.81	3.72	3.53	3.45	3.11
**Cumulative contribution rate / %**	19.66	37.92	49.52	58.45	65.09	69.90	73.62	77.15	80.60	**83.71**

The cumulative contribution rate means the representativeness of extracted factors for all variables. Generally, 80% is regarded as the critical value. And the larger the value, the stronger the representativeness. The meaning of each principal component is determined by the absolute value of load coefficient. Variables with an absolute value greater than 0.7 can be considered as representative ones of the principal component

As expected, the first principal component (PC1) was the most prominent and accounted for 20% of variation. Traits integrated by PC1 mainly reflected floral organs color (ordered of their importance: petal color at the balloon stage, peduncle color, sepal color, petal outside color, receptacle color, and style color). PC2 interpreted 18% of variation and was influenced by flower diameter, petal length, and petal width, which mainly reflected the flower size. PC3 contributed 12% of the variance mainly through petal length / petal width, petal shape, and petal relative position. PC4 had 9% of the variance, and partially affected petal number per flower and flower type. PC5 explained 7% of variation in the pubescence of floral organs (receptacle pubescence and peduncle pubescence). PC6 (5%) integrated two traits related to the sepal morphology (sepal length / sepal width and sepal apex shape). While the remaining components (PC7: 4%, PC8: 4%, PC9: 3%, and PC10: 3%), were affected by peduncle length, petal surface wrinkle, relative position of stigmas and anthers, and flower shape, respectively.

### Pearson correlation analysis of *Malus* floral phenotypic traits

**Fig. 2:**
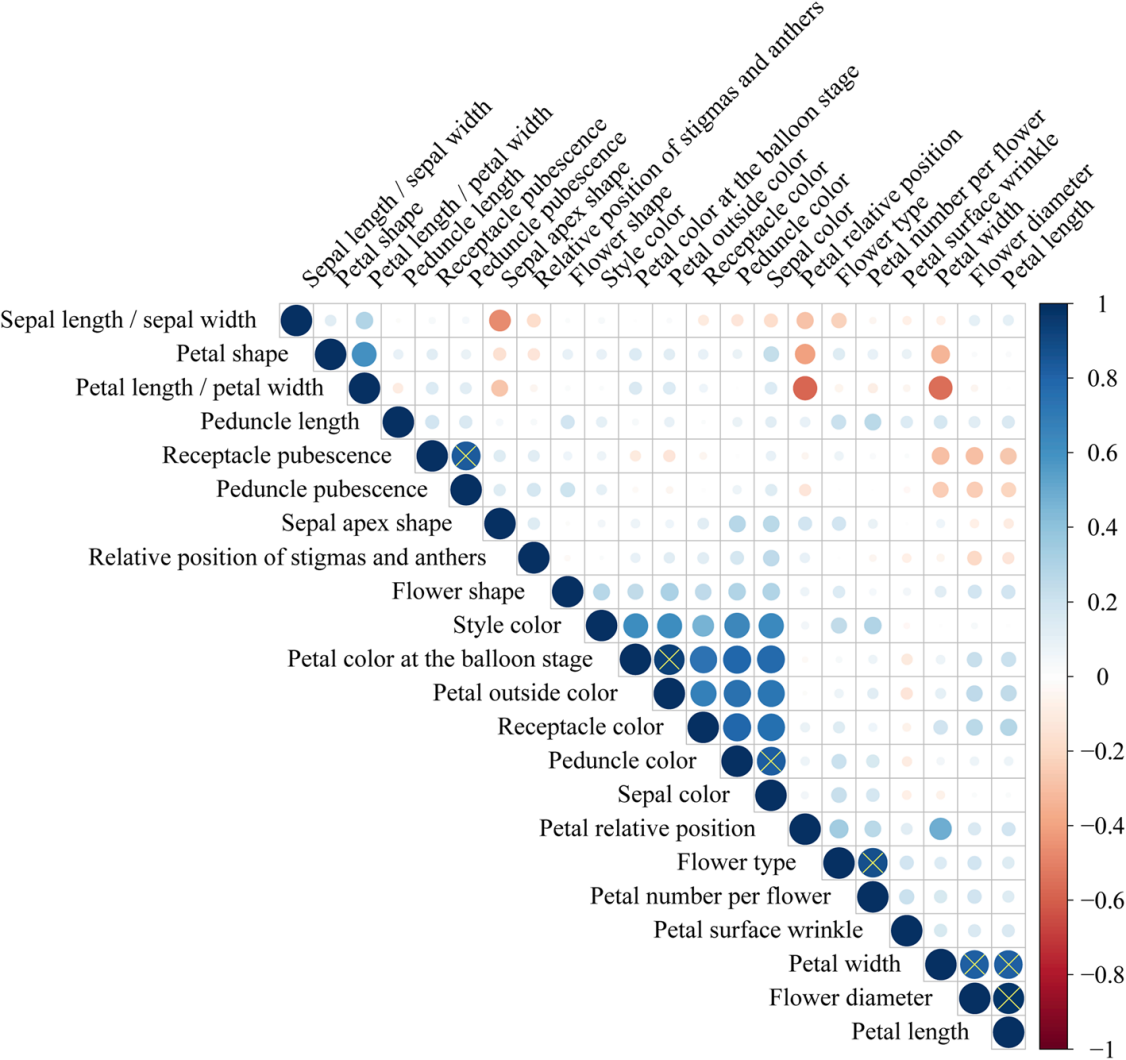
Pearson correlation analysis of *Malus* floral phenotypic traits. The circle marked with ‘×’ represents that the correlation index between phenotypic traits of floral organs is over 0.80 (*r* > 0.80). For these highly relevant traits, we opted to choose either one of the two traits when classifying the taxa

Pearson correlation analysis (taking *r* > 0.80 as the critical value) was performed on 22 floral traits selected by the principal component analysis described above (Fig. 2). It was found that most of the traits were independent of each other, and only few were completely or closely related (e.g., flower diameter and petal length (*r* = 0.98), flower diameter and petal width (*r* = 0.82), petal length and petal width (*r* = 0.81), petal number per flower and flower type (*r* = 0.87), petal outside color and petal color at the balloon stage (*r* = 0.94), peduncle color and sepal color (*r* = 0.83), and receptacle pubescence and peduncle pubescence (*r* = 0.83)). For these highly relevant traits, we opted to choose either one of the two traits for taxa classification. It should be pointed out that although lower correlation coefficient existed between petal shape and petal length / petal width (*r* = 0.61), these two traits were logically related in principle and also in this case, either one could be used.

### Cluster analysis of *Malus* taxa based on important phenotypic traits of floral organs

**Fig. 3:**
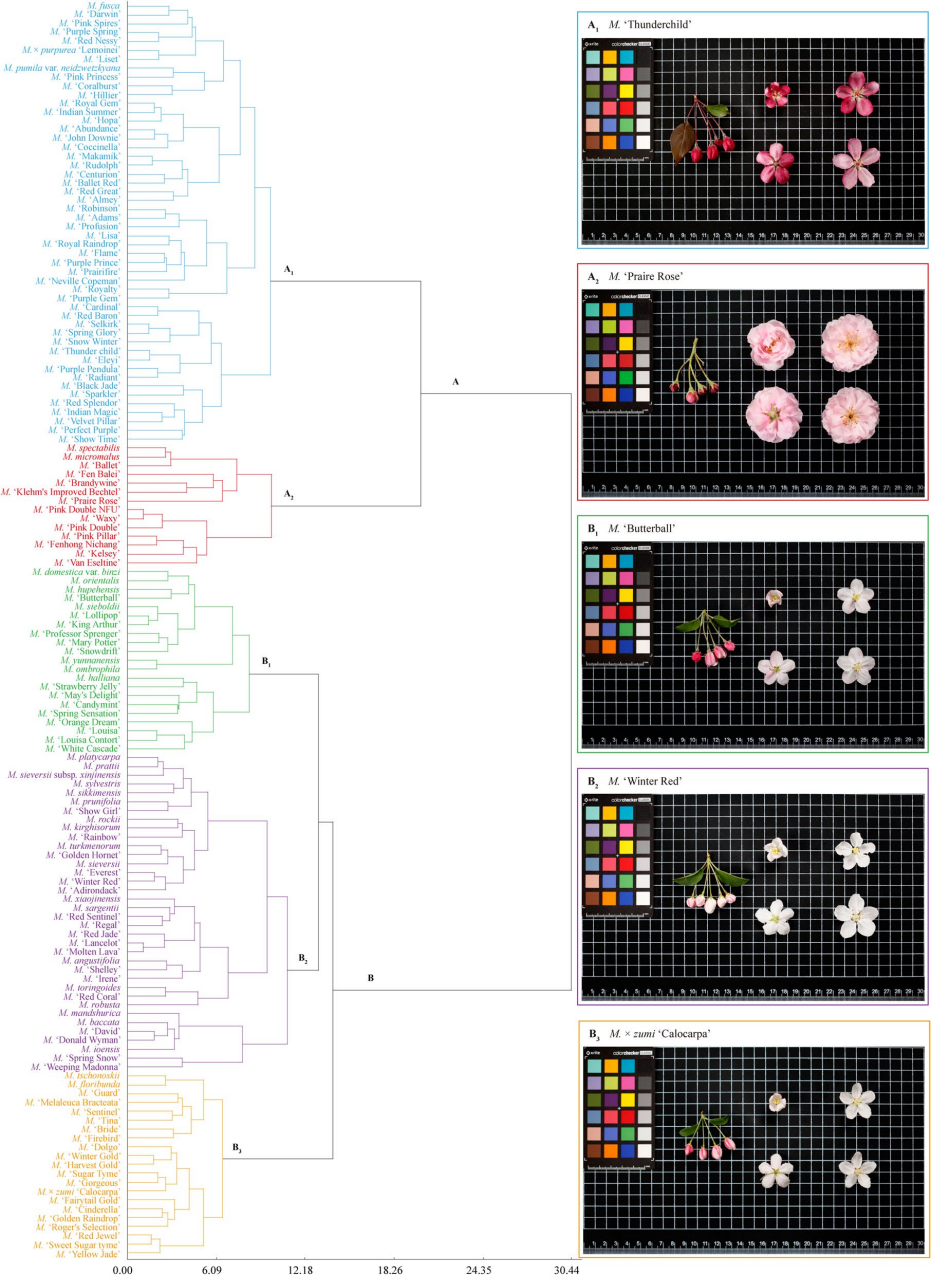
Clustering dendrogram of *Malus* taxa based on important phenotypic traits of floral organs. At Euclidean distances of 21.31 and 11.63, all the 142 taxa were divided into two groups (A, B) and five sub-groups (A_1_, A_2_, B_1_, B_2_, and B_3_), and the characters of floral organs varied significantly between groups and within sub-groups. Taxa belonged to the same sub-groups were labeled with the same color, and the floral organs dynamic map of typical *Malus* taxa for each sub-group was presented in the right rectangular box with corresponding color

Finally, 15 important phenotypic traits of floral organs were selected, that are the peduncle color, petal outside color, receptacle color, style color, flower diameter, petal shape, petal relative position, flower type, peduncle pubescence, sepal length / sepal width, sepal apex shape, peduncle length, petal surface wrinkle, relative position of stigmas and anthers, and flower shape. Figure 3 shows the cluster dendrogram of the studied 142 taxa using flexible-beta method based on these 15 floral traits. At Euclidean distances of 21.31 and 11.63, all taxa could be divided into two groups (A, B) and five sub-groups (A_1_, A_2_, B_1_, B_2_, and B_3_), and the characters of floral organs varied significantly between groups and within sub-groups.

Group A: included a total of 64 taxa (45%) characterized by red flowers and consisted of two sub-groups (A_1_ (50, 35%) and A_2_ (14, 10%)). Taxa in sub-group A_1_ are attractive for their single or semi-double flowers (4.15 ± 0.64 cm in diameter) that are shallow cup-shaped or deep cup-shaped. The petals are red- to dark red-purple, as well as the color of receptacles and peduncles. The relative position of petals is touching or overlapping. Sepal shape of flowers in this sub-group is lanceolate, and their apexes are acuminate in majority. Peduncles are medium in length (2.75 ± 0.75 cm), mostly with no or sparse pubescence. Relative to anthers, stigmas are above or the same height. In sub-group A_2_, the flowers are large (4.87 ± 0.75 cm in diameter), charming with double (15–27 petals), light pink to deep pink, wrinkled petals. The flower shape is flat or deep cup-shaped, and the petals is elliptic. Relative position of petals is overlapping. The sepals are triangular, and their apexes are mostly acute. Peduncles are long (3.16 ± 0.52 cm). In this sub-group, the relative position of stigmas and anthers varied.

Group B: included 78 taxa (55%) and are distinguished by their single, pinkish white or white flowers, gradually changing from pink to rose-red or pure white buds. It contained three sub-groups (B_1_ (21, 15%), B_2_ (36, 25%) and B_3_ (21, 15%). The degree of petal color rhythm (petal color changes during the different flowering stages) of the three sub-groups was B_1_ > B_3_ > B_2_. In sub-group B_1_, taxa are unique for their small (3.44 ± 0.88 cm) flowers that are flat or shallow cup-shaped. Petal shapes are mostly round to ovate, and the petals are overlapping with each other. Flowers in this sub-group have red-purple receptacles, triangular sepals, and acute sepal apexes. The peduncles are medium in length (2.72 ± 0.49 cm), covered with sparse or dense pubescence in its red-purple epidermis. Stigmas are almost of the same height as anthers. In sub-group B_2_, flowers are differed for their medium diameter (4.21 ± 0.73 cm), with flat or shallow cupped, rarely deep cup-shaped colloras. The petal shapes range from ovate to elliptic and their surface is usually wrinkled. Relative position of petals is contacting or overlapping. Peduncles are medium in length (2.69 ± 0.87 cm), covered with no pubescence on their epidermis. Sepal shapes are long lanceolate, and their apexes are acuminate. Receptacles and peduncles color is majorly green or reddish green. As for the sub-group B_3_, flowers are also small in diameter (3.41 ± 0.51 cm), with flat or shallow cupped corollas. The petals are elliptic or narrow elliptic, and their relative position is free or touching. Receptacles and peduncles color is green or reddish green. Thinly or densely hairy are observed in the surface of peduncles. Compared with the other two sub-groups, peduncle length in sub-group B_3_ is relatively shorter (2.43 ± 0.53 cm). The sepals are lanceolate with acuminate apexs. In this sub-group, stigmas are mostly of the same or higher than the anthers height.

### Ancestor-inclined distribution characteristic analysis of *Malus* taxa

**Fig. 4:**
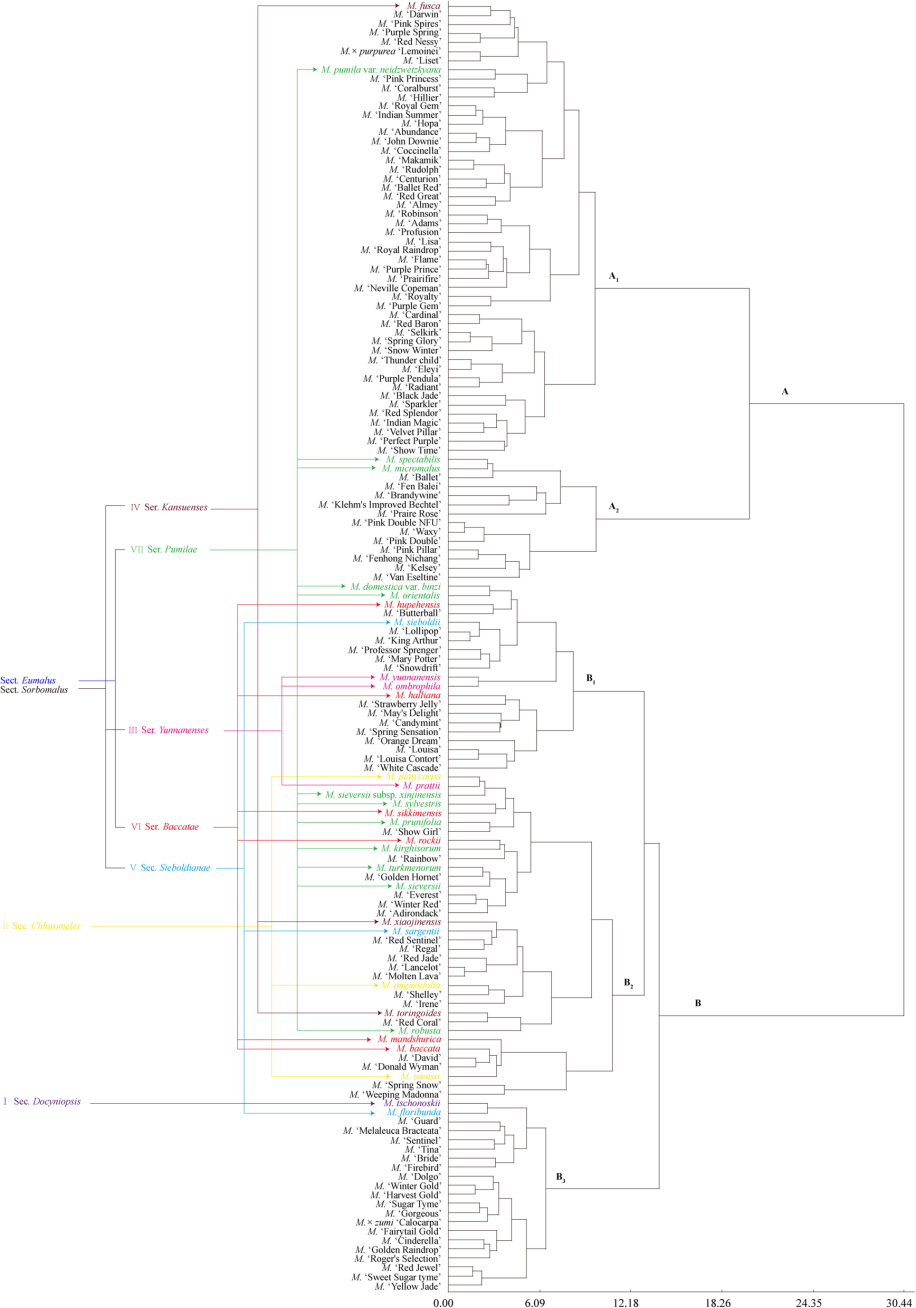
Ancestor*-*inclined distribution characteristics and genetic relationship analysis of *Malus* taxa. According to the *Malus* species taxonomy system proposed by Rehder (1940) [28], Yu et al. (1956) [29], and Li et al. (2001) [30], the 31 species involved in this study belonged to seven sections (series): I, Sect. *Docyniopsis* (one species); II, Sect. *Chloromeles* (three species); III, Ser. *Yunnanenses* of Sect. *Sorbomalus* (three species); IV, Ser. *Kansuenses* of Sect. *Sorbomalus* (three species); V, Ser. *Sieboldianae* of Sect. *Sorbomalus* (three species); VI, Ser. *Baccatae* of Sect. *Eumalus* (six species); and VII, Ser. *Pumilae* of Sect. *Eumalus* (12 species). The order from I to VII corresponded with the sequence of sect/series evolution. Species in the same section (seris) were labeled with the same color. Then the ancestor-inclined distribution characteristics of *Malus* taxa were analyzed from two aspects: species and cultivars, based on the clustering dendrogram of all 142 *Malus* taxa

In this study, ancestor-inclined distribution characteristics of *Malus* taxa were analyzed from two aspects: species and cultivars. In accordance with the *Malus* species taxonomy system proposed by Rehder [28], Yu et al. [29], and Li et al. [30], the 31 species involved in our study belonged to seven sections (series): I, Sect. *Docyniopsis* (one species); II, Sect. *Chloromeles* (three species); III, Ser. *Yunnanenses* of Sect. *Sorbomalus* (three species); IV, Ser. *Kansuenses* of Sect. *Sorbomalus* (three species); V, Ser. *Sieboldianae* of Sect. *Sorbomalus* (three species); VI, Ser. *Baccatae* of Sect. *Eumalus* (six species); and VII, Ser. *Pumilae* of Sect. *Eumalus* (12 species) (Fig. 4). The distribution of *Malus* species belonged to the same section (series) was relatively concentrated. The ancestor-inclined distribution probability reached up to 87% in the two groups and 61% in five sub-groups. From the literature, 33 out of the 111 tested *Malus* cultivars could be completely or partially traced back to their parental taxa (11 species with the floral organ phenotypic data involved in this study) [8–10, 12]. Statistical analysis indicated that the studied 33 cultivars also showed obvious ancestor-inclined distribution characteristics in two groups (A, B) and five sub-groups (A_1_, A_2_, B_1_, B_2_, B_3_), with inclined probability reaching up to 73 and 64%, respectively (Table 2).

**Table 2:**
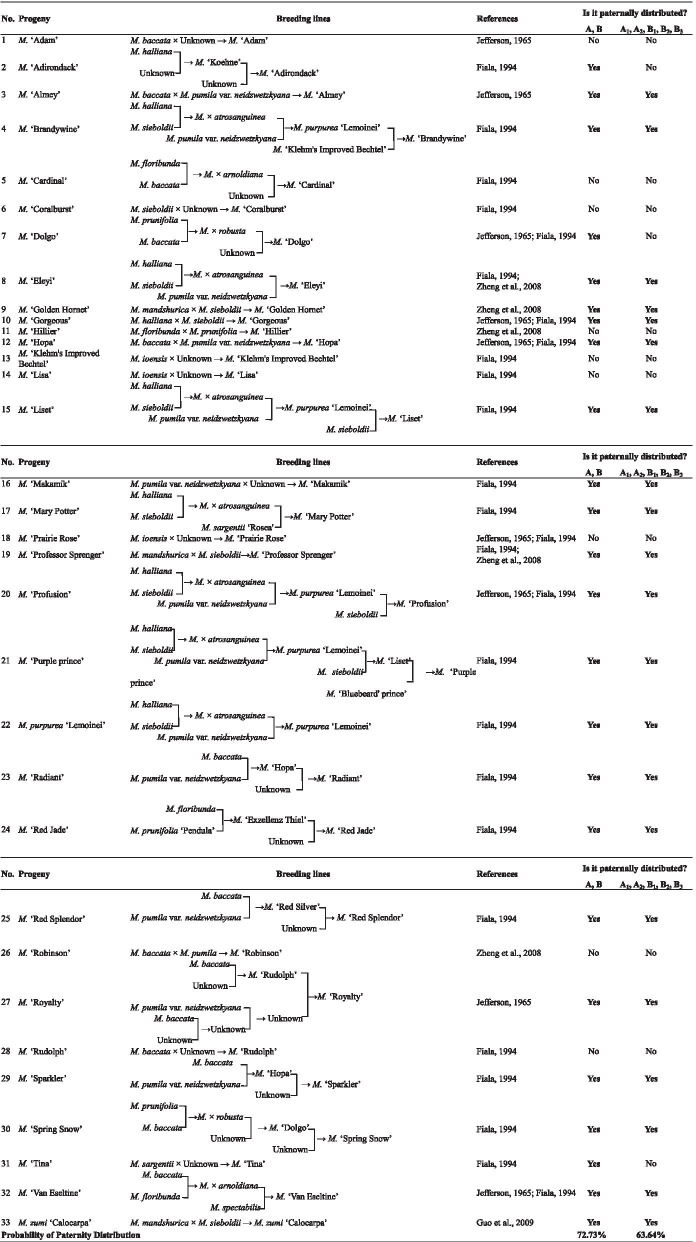
Parents traceability and identification of ancestor-inclined distribution characteristics of *Malus* cultivars

Based on the above distribution characteristics of *Malus* species in the two groups (A, B) and five sub-groups (A_1_, A_2_, B_1_, B_2_, B_3_), it was inferred that the evolutionary order of the four groups/sub-groups (A, B_1_, B_2_, and B_3_) in this study might be: B_3_ → B_2_ → B_1_ → A. According to the order from original to evolved, these four groups/sub-groups and the four sections of *Malus* species in classic taxonomy system were assigned by values: B_3_ (1) → B_2_ (2) → B_1_ (3) → A (4), Sect. *Docyniopsis* (1) → Sect. *Chloromeles* (2) → Sect. *Sorbomalus* (3) → Sect. *Eumalus* (4). It was also found that there was no significant correlation between these two sets of evolutionary data (*R*^2^ = 0.068, *P* = 0.156).

## Discussion

### Establishment of a screening system for *Malus* floral taxonomic traits

Typical angiosperm flowers are composed of sterile sepals and petals and fertile stamens and carpels [31–33]. The significant differences in the number, type, size, shape, color, arrangement, and smell of each part determine its multi-dimensional and complex characteristics [14, 34, 35]. Uni-dimensional variables are usually difficult to describe in its entirety, while the specificity of different groups could be easily masked when several variables were simultaneously considered. Currently, dimensionality reduction of traits is often performed by principal component analysis (PCA) or correlation analysis (R-type cluster analysis), or by artificial screening based on intuitive experience [2, 36, 37]. No systematic and scientific system has been formed for trait screening. In this study, for meeting the requirements of *Malus* taxonomy and aesthetic, a theoretical and technical system (intraspecific uniformity test → interspecific distinctness analysis and one-way ANOVA → principal component analysis → Pearson correlation analysis) was established in accordance with the order of uniformity → distinctness → independence. Twelve qualitative (petal outside color, peduncle color, receptacle color, style color, petal shape, petal relative position, flower type, peduncle pubescence, sepal apex shape, flower shape, petal surface wrinkle, and relative position of the stigmas and anthers) and three quantitative traits (flower diameter, peduncle length, and sepal length/sepal width) were finally extracted, and these traits could reflect most of the information present in the original *Malus* floral dataset. This theoretical and technical trait screening system also has important reference for the extraction of phenotypic characteristics in taxonomy of other ornamental plant resources.

### Taxonomic significance of phenotypic variation of *Malus* floral organs

Floral variation is a positive response of plants to the selection pressure [38–41]. Studies on floral variation not only contributed to our understanding of species evolution [42–45], but also revealed the genetic rules and variation degrees of populations / groups [46–50], which in turn provided a theoretical basis for the protection of species. In this study, through the cluster analysis of the 142 *Malus* taxa based on their floral organs phenotypic traits, we found that the distribution of *Malus* species belonged to the same section (series) was relatively concentrated, with ancestor-inclined distribution probability reaching up to 87% in two groups (A, B) and 61% in five sub-groups (A_1_, A_2_, B_1_, B_2_, B_3_). Among the 33 cultivars that could be traced to all or part of their parents, evident ancestor-inclined distribution characteristics were also observed in the above mentioned groups / sub-groups (the ancestor-inclined distribution probability reaching up to 73 and 64%, respectively). Our results agreed with the classical *Malus* taxonomy system established by Rehder [28], indicating that the phenotypic variation of floral organs could be well applied to the genetic relationship exploration between *Malus* taxa. However, by comparing the evolutionary order of *Malus* sections (Sect. *Docyniopsis* → Sect. *Chloromeles* → Sect. *Sorbomalus* → Sect. *Eumalus*) proposed by Langenfel’D [51] based on classic phenotypic traits with that of *Malus* groups / sub-groups (B_3_ → B_2_ → B_1_ → A) inferred from the cluster dendrogram of the 142 *Malus* taxa, it was found that there was no significant correlation between them (*R*^2^ = 0.068, *P* = 0.156). This indicated that floral variation is unable to reveal the evolutionary relationships of *Malus* species. In fact, the variation or change in different floral organs usually occurs at different taxonomic levels (family, genus, species, ranks below species) [52]. Size, color, smell, and the taste of floral organs are often quite different in species or lower levels [45, 53–55]. Jin [56] concluded that in the taxonomy of subgenus *Tsutsusz* (*Rhododendron*), the tree habit, shape and the size of corollas, could be used to distinguish grades above the species level. The pubescence type of young twigs, number of stamens, size of calyx lobes, pubescence condition of filament or corolla, etc. could be applied in the delimitation of species (taxa below species). In some cases, the pubescence condition of style could be limitedly adopted, while some important traits such as whether stamens are longer than pistils and whether stamens are equal in length should be better avoided. For exploring the evolutionary relationship of *Malus* species based on the phenotypes of floral organs, the screening of taxonomic traits varied at the species level is therefore playing the key role.

## Conclusions

This study innovatively established a scientific system (intraspecific uniformity test → interspecific distinctness analysis and one-way ANOVA → principal component analysis → Pearson correlation analysis) for *Malus* taxonomic traits screening in accordance with the order of uniformity → distinctness → independence. This scientific system also has important reference for the extraction of phenotypic characteristics in taxonomy of other ornamental plant resources. Based on numerical taxonomy, phenotypic variation of *Malus* floral organs was then clearly clarified, as well as its taxonomic significance: Phenotypic variation of floral organs could better explore the genetic relationship between *Malus* taxa. These findings improved our cognition of floral phenotypic variation taxonomic significance under the species level.

## Methods

### Plant materials

A total of 142 *Malus* taxa (including 31 species and 111 cultivars) were collected from the National Repository of *Malus* spp. Germplasm (Yangzhou City, Jiangsu Province, China) (Table 3). All trees were 7 to 10 years old and entered the full blooming phase. Each cultivar was represented by 30 individuals planted with 3 m between rows and 2 m within rows. According to the requirements of randomized block experiment design, 10 plants were taken as one block, and three blocks were set for each taxon.

**Table 3:** List of the 142 taxa collected from the national repository of *Malus* germplasm

No. Species	No. Cultivars	No. Cultivars	No. Cultivars	No. Cultivars
1 *Malus angustifolia*	32 *M.* ‘Abundance’	63 *M.* ‘Harvest Gold’	94 *M.* ‘Professor Sprenger’	125 *M.* ‘Sparkler’
2 *M. baccata*	33 *M.* ‘Adam’	64 *M.* ‘Hillier’	95 *M.* ‘Profusion’	126 *M.* ‘Spring Glory’
3 *M. domestica* var.*binzi*	34 *M.* ‘Adirondack’	65 *M.* ‘Hopa’	96 *M.* ‘Purple Gem’	127 *M.* ‘Spring Sensation’
4 *M. floribunda*	35 *M.* ‘Almey’	66 *M.* ‘Indian Magic’	97 *M.* ‘Purple Pendula’	128 *M.* ‘Spring Snow’
5 *M. fusca*	36 *M.* ‘Ballet’	67 *M.* ‘Indian Summer’	98 *M.* ‘Purple Prince’	129 *M.* ‘Strawberry Jelly’
6 *M. halliana*	37 *M.* ‘Ballet Red’	68 *M.* ‘Irene’	99 *M.* ‘Purple Spring’	130 *M.* ‘Sugar Tyme’
7 *M. hupehensis*	38 *M.* ‘Black Jade’	69 *M.* ‘John Downie’	100 *M.* × *purpurea* ‘Lemoinei’	131 *M.* ‘Sweet Sugartyme’
8 *M. ioensis*	39 *M.* ‘Brandywine’	70 *M.* ‘Kelsey’	101 *M.* ‘Radiant’	132 *M.* ‘Thunderchild’
9 *M. kirghisorum*	40 *M.* ‘Bride’	71 *M.* ‘King Arthur’	102 *M.* ‘Rainbow’	133 *M.* ‘Tina’
10 *M. mandshurica*	41 *M.* ‘Butterball’	72 *M.* ‘Klehm’s Improved Bechtel’	103 *M.* ‘Red Baron’	134 *M.* ‘Van Eseltine’
11 *M. micromalus*	42 *M.* ‘Candymint’	73 *M.* ‘Lancelot’	104 *M.* ‘Red Coral’	135 *M.* ‘Velvet Pillar’
12 *M. ombrophila*	43 *M.* ‘Cardinal’	74 *M.* ‘Lisa’	105 *M.* ‘Red Great’	136 *M.* ‘Waxy’
13 *M. orientalis*	44 *M.* ‘Centurion’	75 *M.* ‘Liset’	106 *M.* ‘Red Jade’	137 *M.* ‘Weeping Madonna’
14 *M. platycarpa*	45 *M.* ‘Cinderella’	76 *M.* ‘Lollipop’	107 *M.* ‘Red Jewel’	138 *M.* ‘White Cascade’
15 *M. prattii*	46 *M.* ‘Coccinella’	77 *M.* ‘Louisa’	108 *M.* ‘Red Nessy’	139 *M.* ‘Winter Gold’
16 *M. prunifolia*	47 *M.* ‘Coralburst’	78 *M.* ‘Louisa Contort’	109 *M.* ‘Red Sentinel’	140 *M.* ‘Winter Red’
*17 M. pumila* var. *neidzwetzkyana*	48 *M.* ‘Darwin’	79 *M.* ‘Makamik’	110 *M.* ‘Red Splendor’	141 *M.* ‘Yellow Jade’
18 *M. robusta*	49 *M.* ‘David’	80 *M.* ‘Mary Potter’	111 *M.* ‘Regal’	142 *M.* × *zumi* ‘Calocarpa’
19 *M. rockii*	50 *M.* ‘Dolgo’	81 *M.* ‘May’s Delight’	112 *M.* ‘Robinson’	
20 *M. sargentii*	51 *M.* ‘Donald Wyman’	82 *M.* ‘*Melaleuca Bracteata*’	113 *M.* ‘Roger’s Selection’	
21 *M.sieboldii*	52 *M.* ‘Eleyi’	83 *M.* ‘Molten Lava’	114 *M.* ‘Royal Gem’	
22 *M. sieversii*	53 *M.* ‘Everest’	84 *M.* ‘Neville Copeman’	115 *M.* ‘Royal Raindrop’	
23 *M. sieversii subsp. xinjinensis*	54 *M.* ‘Fairytail Gold’	85 *M.* ‘Orange Dream’	116 *M.* ‘Royalty’	
24 *M. sikkimensis*	55 *M.* ‘Fen Balei’	86 *M.* ‘Perfect Purple’	117 *M.* ‘Rudolph’	
25 *M. spectabilis*	56 *M.* ‘Fenhong Nichang’	87 *M.* ‘Pink Double’	118 *M.* ‘Selkirk’	
26 *M. sylvestris*	57 *M.* ‘Firebird’	88 *M.* ‘Pink Double NFU’	119 *M.* ‘Sentinel’	
27 *M. toringoides*	58 *M.* ‘Flame’	89 *M.* ‘Pink Pillar’	120 *M.* ‘Shelley’	
28 *M. tschonoskii*	59 *M.* ‘Golden Hornet’	90 *M.* ‘Pink Princess’	121 *M.* ‘Show Girl’	
29 *M. turkmenorum*	60 *M.* ‘Golden Raindrop’	91 *M.* ‘Pink Spires’	122 *M.* ‘Show Time’	
30 *M. xiaojinensis*	61 *M.* ‘Gorgeous’	92 *M.* ‘Praire Rose’	123 *M.* ‘Snowdrift’	
31 *M. yunnanensis*	62 *M.* ‘Guard’	93 *M.* ‘Prairifire’	124 *M.* ‘Snow winter’	

The taxa numbered from 1 to 31 are *Malus* species, and from 32 to 142 are *Malus* cultivars

### Trait measurement, description, and coding

For each cultivar, 10 plants were randomly selected and three consistent, typical and standard full-bloom flowers for each plant were collected, yielding 30 samples in total. All flowers were gathered from the middle of the tree and the branch exposed to the sun. Then, they were placed in a cooler and taken to the laboratory for immediate measurement.

Phenotypic traits evaluation was carried out as recommended by the guidelines for *Malus* distinctness, uniformity and stability test [57] and additional traits were specifically selected for their identification value. All together 44 phenotypic traits of *Malus* floral organs were investigated in this study, including 31 qualitative traits (dimorphic traits and polymorphic traits that can only be observed and present discontinuous variation) and 13 quantitative traits (traits that can be differentiated by quantity and present continuous variation) [58] (Table 4).

**Table 4:** Description and coding of the assessed *Malus* floral phenotypic traits

No.	Phenotypic trait	Trait description (grade) and coding
1	Flower density	Dense (0); Medium (1); Sparse (2)
2	Inflorescence type	Corymbiform (0); Umbellate (1)
3	Buds adhered per inflorescence	No (0); Yes (1)
4	Flower type	Single (0); Semi-double (1); Double (2)
5	Flower shape	Flat (0); Shallow cup (1); Deep cup (2)
6	Flower diameter	Assessed in mm
7	Petal color at the balloon stage	Yellow green (0); White (1); Pinkish White (2); Light pink (3); Deep pink (4); Rose (5); Light red-purple (6); Deep red-purple (7); Deep red (8); Dark red-purple (9)
8	Petal outside color	White (0); Pinkish white (1); Light pink (2); Deep pink (3); Rose (4); Light red-purple (5); Deep red-purple (6); Dark red-purple (7)
9	Petal margin inside color	White (0); Pinkish white (1); Light pink (2); Light red-purple (3); Deep red-purple (4); Dark red-purple (5)
10	Petal center inside color	White (0); Pinkish white (1); Light pink (2); Light red-purple (3); Deep red-purple (4); Dark red-purple (5)
11	Petal base inside color	White (0); Pinkish white (1); Light pink (2); Light red-purple (3); Deep red-purple (4); Dark red-purple (5)
12	Petal relative position	Separated (0); Touching (1); Overlapping (2)
13	Petal number per flower	Counted
14	Petal shape	Circular (0); Oval (1); Ovate (2); Obovate (3); Elliptic (4); Narrow elliptic (5)
15	Petal surface wrinkle	No (0); Yes (1)
16	Petal with prominent veins	No (0); Yes (1)
17	Petal margin with incisions	No (0); Yes (1)
18	Petal length	Assessed in mm
19	Petal width	Assessed in mm
20	Petal length / Petal width	Calculated
21	Claw length	Assessed in mm
22	Sepal color	Green (0); Reddish green (1); Red-purple (2)
23	Sepal apex shape	Acuminate (0); Acute (1)
24	Sepal deflexed	No (0); Yes (1)
25	Sepal pubescence	Dense (0); Sparse (1); None (2)
26	Sepal length	Assessed in mm
27	Sepal width	Assessed in mm
28	Sepal length / Sepal width	Calculated
29	Receptacle color	Green (0); Reddish green (1); Red-purple (2)
30	Receptacle pubescence	Dense (0); Sparse (1); None (2)
31	Relative length of sepals and receptacle	Longer (0); Same length (1); Shorter (2)
32	Peduncle habit	Upright (0); Drooping (1); Weeping (2)
33	Peduncle color	Green (0); Reddish green (1); Red-purple (2)
34	Peduncle pubescence	Dense (0); Sparse (1); None (2)
35	Peduncle length	Assessed in mm
36	Peduncle thickness	Assessed in mm
37	Pistil number per flower	Counted
38	Style color	Light green (0); Yellow green (1); Light red-purple (2); Deep red-purple (3)
39	Style base with pubescence	No (0); Yes (1)
40	Stamen number per flower	Counted
41	Anther with red-purple membrane	No (0); Yes (1)
42	Anther color	White (0); Light yellow (1); Yellow (2); Orange (3)
43	Filament color	White (0); Light red-purple (1); Deep red-purple (2)
44	Relative position of stigmas and anthers	Below (0); Same level (1); Above (2)

Qualitative traits were directly observed in the field, and the final values of quantitative traits were calculated as the mean value of 30 replicates. Hierarchical number coding system was applied for the qualitative traits following the order from ancestral to evolutionary as far as possible. Consecutively arranged non-negative integers 0, 1, 2, 3, ..., were taken for expression. The dimorphic traits with an evolutionary relationship that was difficult to determine were generally coded as 1 (Yes) and 0 (No). No coding was applied for the quantitative traits and the mean values of the 30 replicates were directly used for further analysis

For four consecutive years (2017 to 2020: end of March to mid-April or late April), the 44 traits were repeatedly assessed for correction. Qualitative traits were directly observed in the field, and the final values of quantitative traits were calculated as the mean value of 30 replicates. Hierarchical number coding system was applied for the qualitative traits following the order from ancestral to evolutionary as far as possible. Consecutively arranged non-negative integers 0, 1, 2, 3, ..., were taken for expression. The dimorphic traits with an evolutionary relationship that was difficult to determine were generally coded as 1 (Yes) and 0 (No) [59, 60]. No coding was applied for the quantitative traits and the mean values of the 30 replicates were directly used for further analysis (Table 4).

### Screening of taxonomic traits

**Fig. 5:**
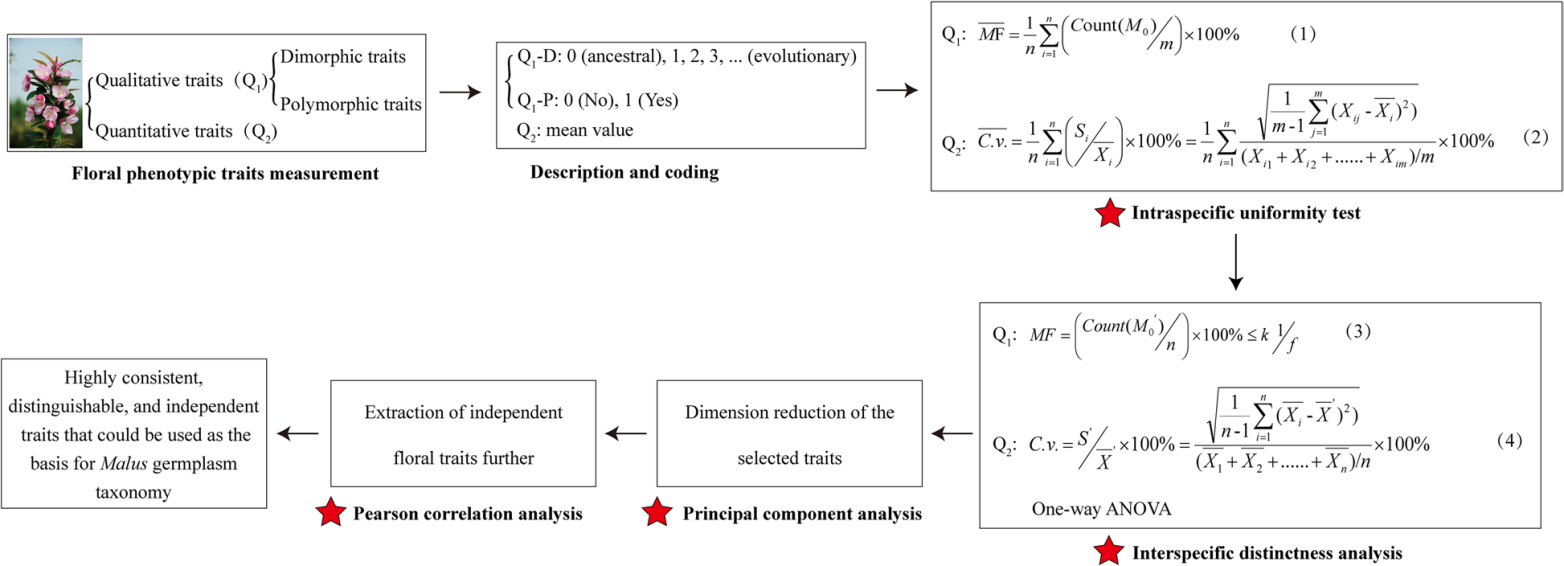
The floral trait screening system for *Malus* taxonomy. The floral trait screening system mainly consists of four steps: intraspecific uniformity test, interspecific distinctness analysis, principal component analysis and Pearson correlation analysis. The intraspecific uniformity of qualitative and quantitative traits was expressed by the mean mode frequency ($\overline{MF}$) and the mean coefficient of variation ($\overline{C.v.}$), respectively. And the interspecific distinctness analysis of qualitative traits was measured by the closeness of the mode frequency (*MF*) and theoretical frequency (${1}\left/{f}\right.$) of the rank that each trait shows in all taxa, while of quantitative traits was measured by the coefficient of variation (*C*. *v*.)

To obtain the traits that are highly consistent, distinguishable, and independent, a scientific system for *Malus* taxonomic traits screening was established (Fig. 5).

#### Intraspecific uniformity test

The intraspecific uniformity test for qualitative traits is expressed by the mean mode frequency ($\overline{MF}$), and for quantitative traits is expressed by the mean coefficient of variation ($\overline{C.v.}$). If $\overline{MF}$≥90% or $\overline{C.v.}$≤10%, then the qualitative (quantitative) trait has met the uniformity requirements.

1 $$\overline{MF}=\frac{1}{n}\sum \limits_{i=1}^n\left({Count\left({M}_0\right)}\left/{m}\right.\right)\times 100\%$$

2 $$\overline{C.v.}=\frac{1}{n}\sum \limits_{i=1}^n\left({{S}_i}\left/{\overline{X_i}}\right.\right)\times 100\%=\frac{1}{n}\sum \limits_{i=1}^n\frac{\sqrt{\frac{1}{m-1}\sum \limits_{j=1}^m{\left({X}_{ij}-\overline{X_i}\right)}^2\Big)}}{\left({X}_{i1}+{X}_{i2}+\dots \dots +{X}_{im}\right)/m}\times 100\%$$

where *n* denotes the number of taxa; *m* denotes the number of repetitions; *M*_0_, *S*_*i*_ and $\overline{X_i}$ denotes the rank with highest frequency of occurrence, standard deviation of the observed values, and the mean observed values of each trait in each taxa’s *m* repetitions, respectively.

#### Interspecific distinctness analysis

The interspecific distinctness analysis of qualitative traits is simply measured by the closeness of the mode frequency (*MF*) and theoretical frequency (${1}\left/{f}\right.$) of the rank that each trait shows in all taxa. If $MF\le{k}\,{1}\left/{f}\right.$, the qualitative trait is more differentiated among all taxa. For quantitative traits, it is expressed by the coefficient of variation (*C*. *v*.) of the mean value of each trait in all taxa. If *C*. *v*.≥15%, it was considered that the differentiation degree of this trait is high among all the taxa. One-way ANOVA (Tukey’s method) should be performed on quantitative traits as well.

3 $$MF=\left({Count\left({M_0}^{\prime}\right)}\left/{n}\right.\right)\times 100\%\le k\,{1}\left/{f}\right.$$

4 $$C.v.={{S}^{\prime}}\left/{{\overline{X}}^{\prime}}\right.\times 100\%=\frac{\sqrt{\frac{1}{n-1}\sum \limits_{i=1}^n{\left(\overline{X_i}-{\overline{X}}^{\prime}\right)}^2\Big)}}{\left(\overline{X_1}+\overline{X_2}+\dots \dots +\overline{X_n}\right)/n}\times 100\%$$

where, *M*_0_^′^ denotes the rank of each trait that appears the most in all taxa; *k* is a coefficient that depends on the number of ranks (*f*) of each trait appeared in all taxa; *S*^′^ and ${\overline{X}}^{\prime }$, respectively, denote the standard deviation and the average of observed mean values of each trait in all taxa.

#### Principal component analysis and Pearson correlation analysis

On the premise of higher uniformity and distinctness, principal component analysis (PCA) and Pearson correlation analysis were used to further reduce the dimensionality of the selected traits. In order to eliminate the impact of different dimensions on data analysis, the standard deviation (STD) normalization process was performed in advance on the original numerical matrix; that is, the orthonormal process.

### Cluster analysis and ancestor-inclined distribution characteristics of *Malus* taxa

Based on the extracted taxonomic traits that could reflect the phenotypes of floral organs, the 142 taxa were quantitatively classified using flexible average method so as to reveal the phenotypic diversity of *Malus* floral organs. And meanwhile, the ancestor-inclined distribution characteristics was analyzed from two aspects: species and cultivars, aiming at clarifying *Malus* floral variation taxonomic significance.

### Data processing

Origin 9.0, DPS 9.5, R 3.6.1, and Adobe Illustrator CS5 were used for data processing and graph plotting.

## Data Availability

A total of 142 *Malus* spp. germplasms (including 31 species and 111 cultivars) were collected from the National Repository of *Malus* spp. Germplasm (Yangzhou City, Jiangsu Province, China). The datasets used and analysed during the current study could be available from the corresponding author on reasonable request.
